# Characterization of biomechanical risk factors during endoscopic submucosal dissection: Ergonomic pilot study

**DOI:** 10.1055/a-2655-1195

**Published:** 2025-08-01

**Authors:** Clara Yzet, Léa Leroy, Sylvain Chamot, Mathieu Pioche, Franck Brazier, Jean-Phillippe Le Mouel, Jérôme Rivory, Romain Gerard, Alexandru Lupu, Julien Branche, Stéphane Delanaud, Mathurin Fumery, Frederic Telliez

**Affiliations:** 1 36673 Gastroenterology, Amiens-Picardy University Hospital, Amiens, France; 2 41666 Regional Center for Occupational and Environmental Diseases of Hauts-de-France, Amiens-Picardy University Hospital South Site, Amiens, France; 3 Gastroenterology, Edouard Herriot Hospital, Lyon, France; 4 26902 Gastroenterology, CHRU de Lille, Lille, France; 5 Gastroenterology and Endoscopy, Pavillon L Edouard Herriot Hospital, Lyon, France; 6 26902 Gastroenterology, Lille Regional and University Hospital, Lille, France; 7 26993 Laboratoire PériTox UMR_I 01, Institut d’Ingénierie de la Santé-UFR Médecine, Université de Picardie Jules Verne, Amiens, France

**Keywords:** Quality and logistical aspects, Training, Quality management, Endoscopy Lower GI Tract, Endoscopic resection (polypectomy, ESD, EMRc, ...)

## Abstract

**Background and study aims:**

Musculoskeletal disorders (MSDs) are prevalent among endoscopists. The aim of this study was to evaluate biomechanical risk factors for MSDs in gastroenterologists performing ESD.

**Methods:**

An observational study was performed among interventional endoscopists performing ESD in three French centers. Physical constraints were assessed using an analog scale of perceived physical effort intensity and physiological sensors to measure joint angulation kinematics and muscle activity levels (flexor and extensor carpi radialis muscles) during diagnostic colonoscopy and ESD. High muscle strain was defined as any muscle activation exceeding 10% of maximum voluntary contraction (MVC). Two distinct sub-tasks of ESD were identified: lesion marking and circumferential incision phase (ESD-1) and dissection phase (ESD-2).

**Results:**

Six interventional gastroenterologists participated in the study. Perceived physical effort was significantly greater for ESD compared with colonoscopy ( *P* = 0.03). Time spent at more than 10% MVC for the right extensor carpi radialis was significantly higher during ESD-1 than during colonoscopy (+15%, *P* = 0.04). The greatest strain was observed in the left extensor carpi radialis. This muscle was particularly exposed because more than 50% of the time was spent at more than 10% of MVC during colonoscopy and up to more than 80% during ESD-1 and -2. Time spent in the neck flexion risk zone was significantly higher during ESD-2 than during colonoscopy (+42%, *P* = 0.046).

**Conclusions:**

ESD increased the risk of musculoskeletal strain. It is crucial to develop prevention programs to reduce risk of MSD in the population of gastroenterologists.

## Introduction

Prevalence of musculoskeletal disorders (MSD) among endoscopists is estimated to range from 29% to 89%, leading to health issues for practitioners and negatively impacting procedure safety and performance during procedures 1 2 3 4 5 6 7 8 9 10 11 .

Factors associated with MSDs among endoscopists include a high number of procedures (over 20/week), prolonged procedure times (over 16 hours/week), the age of the endoscopist (>40 years vs ≤ 40 years), and female sex 3 5 6 7 9 12 13 . Awkward postures and invariability of the precise gesture are biomechanical risk factors of MSD.

Evolution of interventional digestive endoscopy, especially with introduction of endoscopic submucosal dissection (ESD), has led to increased procedure complexity characterized by an increase in procedure duration, greater precision of the task, and maintenance of prolonged static posture. The spread of ESD techniques in endoscopic procedures may elevate risk of MSDs due to longer procedure time 14 15 16 17 .

Recommendations have been proposed to enhance practitioner comfort, akin to those utilized by digestive surgeons 18 19 . These include utilizing a height-adjustable examination table, positioning the screen at eye level, and using a seat during procedures.

Most of the studies on endoscopist MSDs are based on surveys, which introduce biases when quantifying the role of endoscopic procedures in development of MSDs. Recently, Shergill et al. demonstrated that during colonoscopy, left wrist extensor muscle activity exceeded established thresholds with the greatest risk of constraints occurring during colonic insertion 20 .

Still, there are limited data on biomechanical stresses encountered by endoscopists during many specific procedures (endoscopic retrograde cholangiopancreatography, endoscopic mucosal resection) and particularly ESD. Quantitative assessment of muscular and postural strains associated with ESD procedure activity and tools is crucial to evaluate them against risk thresholds and to prevent MSD occurrence.

The goal of this study was to assess, for the first time, biomechanical risk factors such as muscular, kinematic angulation and awkward posture strains during ESD and to compare them with strains of diagnostic colonoscopy.

## Methods

### Population

Interventional endoscopists from three French university hospital (Amiens, Lyon, Lille) were recruited to participate in 2024. The endoscopists provided informed consent. The study was approved, according to national guidelines, by the CNIL committee (Comité Consultatif sur le Traitement de l’Information en matière de Recherche dans le domaine de la Sante) - record number NCT06549894. Each gastroenterologist completed a survey collecting demographic information such as sex, age, body mass index, years of endoscopy practice. Type of colonoscope used, procedure duration, use of a height-adjustable examination table, and positioning of the screen at eye level were noted. Participants performed one diagnostic colonoscopy and one ESD after placement of biomechanical measurement devices. To increase accuracy and because we suspected that gesture and therefore biomechanical strain were different, the ESD procedure was split in two specific sub-tasks: the initial phase of incision to access to the submucosal space (ESD-1) and the second phase of submucosal dissection (ESD-2).

### Outcome measures

#### Perceived physical effort or discomfort

To assess physical effort or discomfort during the different endoscopy procedures, physicians were asked to complete a Borg scale 21 . The 10-point rating scale was used (0 = no effort, 10 = maximal effort).

#### Forearm muscle activity

**Fig. 1:**
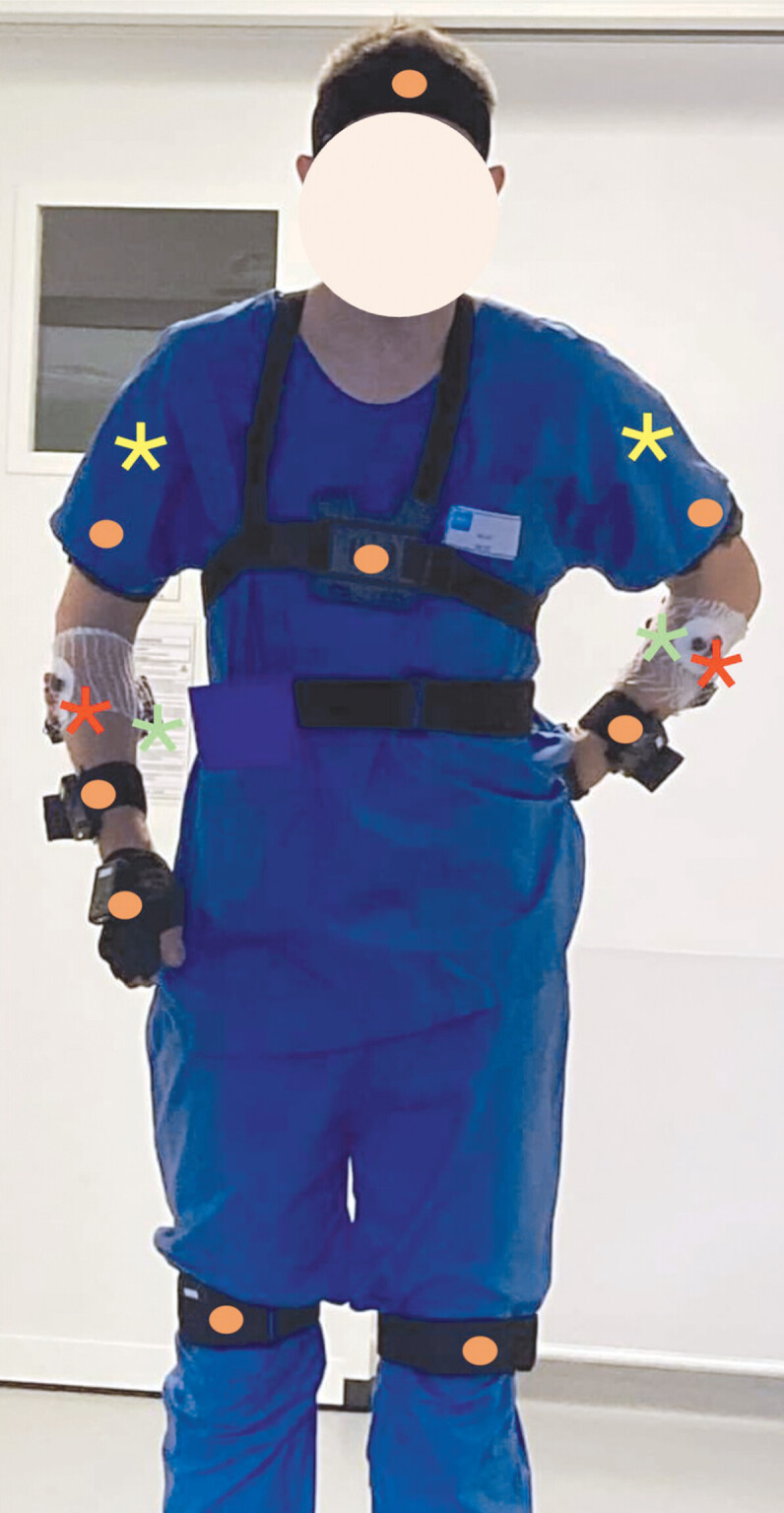
Schematic representation of electromyographic and angular sensor positions Yellow stars: deltoid EMG sensor; red star: flexor carpi radialis; green star: extensor radi carpialis; orange circle: angular sensor for neck, trunk, shoulder, elbow and wrist.

**Fig. 2:**
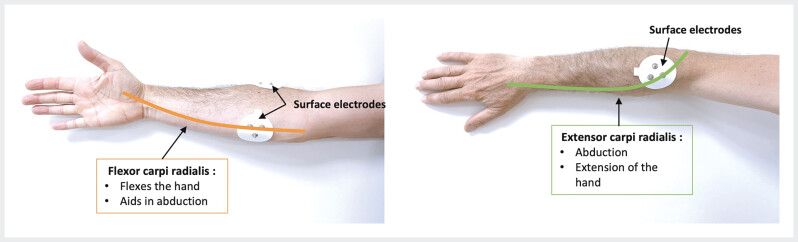
Schematic representation of flexor and extensor carpi radialis.

A wireless transmission module T-Sens was used to measure bilateral muscle surface electromyographic (EMG) activity of the common flexor and extensors carpi radialis. Bipolar surface electrodes were placed on the skin after abrasion and cleaning with alcohol (EMG Triode Electrode, Thought Technology Ltd) using standard anatomic locations ( Fig. 1 and Fig. 2 ). Root mean square value of EMG was directly collected at a sampling rate of 128 Hz and was calculated and smoothed with a time window of 300 ms.

Three maximum voluntary contractions (MVCs) for each muscle were elicited for 3 to 5 seconds by maximally resisting appropriate joint motions 22 . The signal for each muscle was then normalized to %MVC.

#### Head, neck, back and upper-limb joint positions and movements

11 Inertial Measurement Units (IMU) T-Sens (T-Sens Motion, TEA Ergo, France) were used to record position of the different joints and to record 3 degrees of freedom of the upper-limb movements during endoscopy procedure ( Fig. 1 ). T-Sens wireless motion sensors integrate a 3-axis accelerometer, a 3-axis gyroscope, and a 3-axis magnetometer (IMU characteristics: precision head/pitch, roll: 2°/0.5°; gyroscope: ± 2000°/s; accelerometer ±16 g; magnetometer ± 2.5 Gauss). They were attached to the hands, forearms, arms, head, trunk, low back and thighs. Their acquisition frequency was 32 Hz. Using the manufacturer's software, the IMUs were automatically calibrated to similar parameters landmark. At the end of the acquisitions, the software automatically generates processed data on the selected joints. The data is then streamed into CAPTIV (TEA Ergo, France), a software that enables accurate displays and analysis of human body motion angles on joints, angular speed and acceleration.

#### Biomechanical parameters

**Muscle activity**

Muscle activity was expressed as %MVC. High muscle strain of muscle groups was defined as any contraction of the muscles more than 10% of the maximum voluntary contraction which allows to determine the time spent over this threshold 23 .

**IMU measures**

For neck, trunk, and wrist postures, angles were reported as flexion/extension in the sagittal plane. For the shoulders and elbow, angulation was reported with 0° being at the neutral position of the arm. Posture angles were measured during a static endoscopic task. Static posture is defined as joint angular velocity lower than 1°/s 24 25 . Percent time spent in demanding postures was calculated. Such postures were defined as angles outside the range of the recommended limits 26 27 28 : >10° neck flexion or extension, >20° trunk flexion, >45° shoulder elevation, <60° or >100° elbow flexion (forearm position), >0° wrist flexion or >30° wrist extension. Time in demanding postures was compared between the different operating procedures.

### Statistical analysis

Due to the low number of participants, non-parametric statistic tests were performed and data were expressed with the median (min-max). All metrics were compared between three tasks: colonoscopy, the ESD first phase (ESD-1): injection and incision of the mucosa and the second phase of ESD (ESD-2): dissection, using non-parametric, paired Wilcoxon test. Effect size between endoscopic procedures was calculated using r coefficient. Effect size r was calculated as Z statistic divided by square root of the sample size (N). Interpretation of effect size was: small (r = 0.1), medium (r = 0.3), large (r ≥ 0.5).

## Results

### Population

Six endoscopists were included in this study. The majority were men (83.3%) with a median age of 40 years (37.8–40) and practice of endoscopy for 11 years (8–32) ( Table 1 ).

**Table:** **Table 1** Gastroenterologist characteristics.

	**N = 6**
Men, n (%)	5 (83.3)
Age, years, median (IQR)	40 (37.8–40)
Body mass Index, kg/cm ^2^ ) median (IQR)	21.9 (21.1–23.6)
Years of endoscopy practice, median (IQR)	10.8 (6.4–11.8)
Right-handed, n (%)	5 (83.3)
Musculoskeletal disorders, n (%)	6 (100)
Neck	3 (50)
Shoulders	1 (16.7)
Elbow	2 (33.3)
Wrist	3 (50)
Back	5 (83.3)
Hip	2 (33.3)
Knee	1 (16.7)
Ankle	0 (0)
Hours spent in endoscopy/week, median (IQR)	24.3 (22.9–26.3)
IQR, interquartile range.	

Data were fully available for 12 procedures (6 colonoscopies, 6 ESD). Median time for a colonoscopy was 9 minutes (7–64) and 43 minutes (14–98) for ESD ( *P* = 0.01). ESD-1 lasted 19 minutes (18–44) and ESD-2 lasted 24 minutes (9–77) ( *P* = 0.35).

### Perceived physical effort

Perception of physical effort was significantly greater for ESD than for colonoscopy (median Borg of 3.5 [3.0–4.8] vs 2 [.2–2.0]; *P* = 0.03).

### Muscle activation

**Fig. 3:**
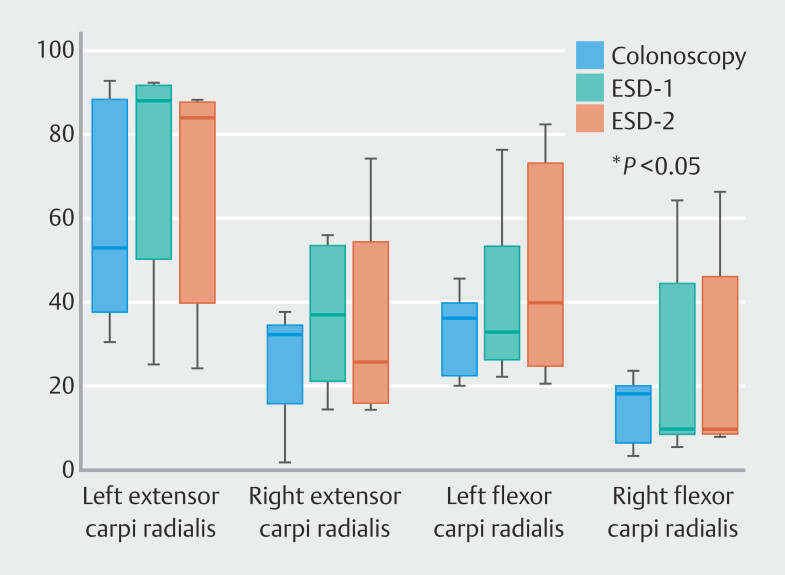
Mean time spent >10% of maximum voluntary contractions for right and left extensor and flexor carpi radialis during colonoscopy and ESD.

Table 2 presents levels of muscle activation during the procedure. Levels of muscle activation during ESD-1 and -2 were not significantly different from muscle activation measured during colonoscopy. Only time spent >10% MVC for right extensor carpi radialis during ESD-1 procedure was significantly higher (+15%) than during colonoscopy ( Fig. 3 ). No difference was observed with ESD-2. The effect size was r = 0.90, which demonstrates a huge effect.

**Table:** **Table 2** Muscle activation levels (%MVC) (median, min-max) during different endoscopic procedures.

	Colonoscopy	ESD-1	ESD-2	*P* value
Right flexor carpi radialis	5.5 (2.5–6.9)	3.8 (2.9–16)	3.7 (2.9–21)	NS
Left flexor carpi radialis	8.9 (4.8–11.8)	9.0 (4.6–15.3)	10.3 (4.4–17.5)	NS
Right extensor carpi radialis	7.9 (3.7–9.0)	10.0 (5.8–13.0)	7.3 (4.1–18.6)	NS
Left extensor carpi radialis	11.8 (8.0–25.2)	19.6 (7.5–39.5)	18.4 (8.7–26.4)	NS
ESD-1, endoscopic submucosal dissection phase 1; ESD-2, endoscopic submucosal dissection phase 2; %MVC, % of maximal voluntary contraction; NS, nonsignificant.				

Value of time spent >10% MVC measured for the left extensor carpi radialis for all the operating procedures was higher than 50% of time. These values were increased during the ESD-1 and ESD-2 procedures vs colonoscopy (+ 67% and +59%, respectively) without reaching statistical difference.

### Posture and angulation during the endoscopic procedures

Table 3 presents posture angles measures, and the statistical difference of percent time spent in demanding posture in the different conditions.

**Table:** **Table 3** Percent time spent in demanding posture (median (min–max)) (as defined in Methods) during different endoscopic procedures.

	Colonoscopy	ESD-1	ESD-2	Comparison	P value	r (effect size)
Neck flexion	27.0 (15.6–93.3)	22.2 (13.4–61.1)	37.9 (25.2–72.9)	Co vs ESD-2 ESD-1 vs -2	*P* = 0.046 *P* = 0.028	0.81 0.89
Trunk flexion	0.09 (0–48.8)	8.1 (0–26.0)	5.8 (0–24.8)		NS	
Right shoulder flexion	0.9 (0–28.6)	1.1 (0.21–3.6)	2.2 (0.05–6.1)		NS	
Left shoulder flexion	0.04 (0–37.6)	0.1 (0.03–0.9)	0.2 (0–24.4)		NS	
Right elbow (<60°, see Methods)	80.6 (52.9–89.7)	66.7 (12.4–70.0)	68.9 (54.5–74.6)	Colonoscopy vs ESD-1	*P* = 0.028	0.89
Left elbow (>100°, see Methods)	19.3 (3.6–69.0)	47.0 (0.8–92.2)	83 (0.86–95.8)	Colonoscopy vs ESD-2	*P* = 0.046	0.81
Right wrist (extension)	41.2 (21–56.1)	40.9 (24.0–78.2)	70.3 (54–82.6)		NS	
Left wrist (extension)	43.1 (9.7–87.1)	54.2 (12.4–94.9)	50.6 (5.9–89.0)	Colonoscopy vs ESD-1 ESD-1 vs ESD-2	*P* = 0.028 P = 0.046	0.89 0.81
ESD-1, endoscopic submucosal dissection phase 1; ESD-2, endoscopic submucosal dissection phase 2; NS, nonsignificant.						

Neck demanding flexion posture is significantly increased during ESD-2 vs the other operating procedures ( *P* = 0.046). For trunk and shoulder, time spent in demanding postures seemed relatively low and was not different whatever the endoscopic procedures. For the other joints, time spent in angles outside of the recommended range was from 19.3% to 83%.

Independently on the endoscopic procedure, right elbow (forearm position) angle was lower than 60° and left elbow angle was higher than 100°. Time in demanding posture for right elbow was significantly higher during colonoscopy than during ESD-1. For left elbow, the constraint was higher during ESD-2 than during colonoscopy ( *P* = 0.046).

For all three endoscopic procedures, left and right wrist position were in extension demanding posture and lasted more than 40% of time. The most important percentage of time spent in a demanding posture was for the right wrist during the ESD-2 procedure, although the differences with the other endoscopic procedures were not significant. In contrast, percentage of time spent in a demanding posture for the left wrist was significantly higher during ESD-1 than during the other two endoscopic procedures.

## Discussion

In this prospective pilot study, we showed that endoscopy is associated with biomechanical stresses whatever the typology of technique for diagnostic or therapeutic means.

First, this study revealed that perception of physical effort using the Borg scale was significantly greater for ESD compared with diagnostic colonoscopy even if the level of exertion perceived during colonoscopy and ESD remain moderate for highly trained physician. We further analyzed biomechanical stresses by measuring forearm muscle activity and joint strain using IMU sensors. We have pointed out that except for the right elbow, most of the biomechanical parameters show that the level of strain is even greater in ESD than in colonoscopy. Indeed, the strain is considerable for all the forearm muscles, with more than 25% of the time spent higher than 10% of the MVC regardless of type of endoscopy. Right extensor carpi radialis muscle activity was more important during the ESD-1 procedure than during classic colonoscopy, which introduces risk of even more musculoskeletal injuries, such as epicondylitis.

The greatest strain seems to relate to the left extensor carpi radialis extensors. Shergill et al. showed that the left wrist extensor muscle is intensively used during endoscopy, potentially contributing to risk of MSDs 20 . Our study confirms these results because relative time spent higher than 10% MVC is particularly important, more than 50% for colonoscopy and even more than 80% during ESD-1 and -2 procedures. This could be particularly worrying for the left upper limb because the position of the left forearm is in the risk zone (i.e. with elbow angle higher than 100°) during ESD-1 and mostly during ESD-2 procedures, the biomechanical strain increases at elbow level. The percent time of left wrist in demanding posture is also important during the ESDs procedures, more than 50% of time, and significantly higher during ESD-1 when compared with the two other procedures. To our knowledge, this is the first study to specifically suggest that ESD procedures may be associated with a higher risk of MSDs compared with standard endoscopy. Indeed, most published studies rely on self-reported data, which introduces bias. For example, in 2022, Sturm et al. analyzed responses from 151 gastroenterologists who completed an online questionnaire 1 . In their study, the average number of endoscopic procedures was 86 per week, including only four EMRs and fewer than one ESD per week. Due to the relatively low volume of ESD and EMR procedures, their analysis did not identify any particular type of endoscopic procedure as being more strongly associated with MSDs, despite the clear differences in technical complexity between, for instance, a diagnostic EGD and an interventional ERCP. In contrast, our study specifically focused on ESD, a technically demanding procedure, and highlights its potentially greater biomechanical impact.

In view of these results, the left forearm, therefore, seems particularly exposed to biomechanical risk factors during ESD and particularly during its second phase, which is at least as biomechanically stressful as colonoscopy. Moreover, the position of the neck is also more concerning during ESD-2 sub-task.

Awareness of this topic is important not only for short-term physical comfort, but also can significantly impact the longevity of a provider’s career. Our results provide evidence that muscular constraints may lead to conditions such as epicondylitis, epitrochleitis, carpal tunnel syndrome, tendonitis, and tenosynovitis of the hand and fingers.

The question now is how to prevent this stress. The American Society of Gastroenterology (AGA) has issued good practice recommendations. Working in an environment with a temperature of 19°C, soft lighting, and adjustable screens and tables is advised 18 . However, the key factor leading to disorders is repetition and duration of tasks.

Further strategies to mitigate these risks need to be developed and implemented. First, reducing the length of procedures could help to minimize risk of developing MSDs. While shortening the duration of colonoscopies may not be feasible, because a minimum time is required to ensure quality, progress has been made in reducing the time required for ESD procedures. This reduction is largely due to practitioner experience and adoption of traction strategies, leading to a significant decrease in procedure time in recent years 14 16 29 . Teamwork with other physicians to split the longer procedure is also a way. Second, the advent of robot-assisted surgeries has demonstrated potential in reducing postoperative discomfort and muscle strain in the upper extremities, particularly on the dominant side of the surgeon 30 31 . However, this advancement comes with its own set of challenges, including increased static neck positioning and subjective back stiffness 32 . This highlights the importance of continuous development in ergonomics and physical and occupational therapy interventions aimed at reducing MSD and enhancing surgeon longevity. In addition, use of exoskeletons has also emerged as a promising option, with studies indicating a reduction in fatigue without negatively impacting surgical procedures 33 34 35 . In that regard, Shergill et al. (2021) have evaluated an endoscope assist device (antigravity support arm) during a simulated task and shown a small reduction of 1.6% MVC in the left extensor carpi radialis muscle activity.

Integration and development of such innovative tools and techniques could pave the way for more sustainable practices in interventional endoscopy and surgery, ultimately improving the well-being and efficiency of healthcare professionals. In light of our results, it seems urgent to consider procedure time and frequency to mitigate risk of developing MSDs of the left arm. This risk appears to be even higher for doctors performing ESD. If robotization strategies are not feasible now, the question remains how to better organize operational planning and increase the number of trained doctors to help reduce incidence of MSDs.

We are aware that our study has some bias. First, the number of subjects evaluated was low and the endoscopist population was homogeneous, which prevented a meaningful analysis of the impact of endoscopic experience on biomechanical measurements. Second, our study did not evaluate psychological stress generated by the procedure, but it can be assumed that ESD increases this type of stress due to the higher risk of perforation and bleeding complications compared with colonoscopy, which in turn could increase risk of MSDs. Further studies must be conducted to identify, assess, and prevent psychosocial risk factors related to endoscopy activity and notably ESD procedures. Finally, we did not monitor all the muscles, we focused on the extensor and flexor carpi radialis for EMG measurement. To minimize potential bias related to fatigue or cumulative workload, recordings were conducted in the morning during the first colonoscopy or ESD procedure of the day. The choice of these muscles is due to their continuous use during endoscopic procedures: pressing valves and switches, rotating the wheels, holding the endoscope, and applying forces/torques to the insertion tube.

## Conclusions

To conclude, although ESD offers significant benefits in treating complex gastrointestinal lesions, it also introduces substantial biomechanical strain, increasing long-term risk of MSDs among ESD practitioners.

It is, therefore, essential to develop prevention programs, such as education and ergonomics training, to benefit the health of endoscopists and quality and safety of care. In addition, development and integration of new devices are essential to combat these issues and enhance the well-being of healthcare professionals.
